# Resilient Consensus Control for Multi-Agent Systems: A Comparative Survey

**DOI:** 10.3390/s23062904

**Published:** 2023-03-07

**Authors:** Jingyao Wang, Xingming Deng, Jinghua Guo, Zeqin Zeng

**Affiliations:** 1 State Key Laboratory of Automotive Simulation and Control, Changchun 130025, China; 2 School of Aerospace Engineering, Xiamen University, Xiamen 361000, China

**Keywords:** multi-agent systems, resilient consensus, secure coordination, DoS attack, spoofing attack, Byzantine attack

## Abstract

Due to the openness of communication network and the complexity of system structures, multi-agent systems are vulnerable to malicious network attacks, which can cause intense instability to these systems. This article provides a survey of state-of-the-art results of network attacks on multi-agent systems. Recent advances on three types of attacks, i.e., those on DoS attacks, spoofing attacks and Byzantine attacks, the three main network attacks, are reviewed. Their attack mechanisms are introduced, and the attack model and the resilient consensus control structure are discussed, respectively, in detail, in terms of the theoretical innovation, the critical limitations and the change of the application. Moreover, some of the existing results along this line are given in a tutorial-like fashion. In the end, some challenges and open issues are indicated to guide future development directions of the resilient consensus of multi-agent system under network attacks.

## 1. Introduction

An agent is an independent individual who can achieve control goals autonomously through environmental perception according to preset knowledge. Usually, an agent only has simple intelligence and basic structure. A multi-agent system (MAS) refers to a networking system composed of a number of intelligent agents who can coordinate and manage through information interaction, so as to achieve complex control objectives that cannot be reached by an agent itself [1]. The distributed consensus control of a MAS is generally to achieve synchronous behavior by constructing a fully distributed controller for each agent. Due to its potential application in broad areas, the consensus control problem has been extensively studied and a lot of significant results have been established in the literature.

In recent years, the coordination control of a MAS has become a hot research topic and has been widely applied in areas such as unmanned aerial vehicle systems, industrial internet of things and wireless sensor networks [2,3,4,5,6,7]. Its research directions mainly include consensus control, formation control and flocking/swarming behavior. As the most fundamental and important topic, consensus has been widely debated and discussed. Consensus requires that a partial state quantity or a full state quantity of agents in a MAS reach an agreement [8,9]. In the ideal case of no interference and attack, there are tremendous amounts of control strategies to enable a MAS to realize the consensus requirements and even to improve the consensus performance [10].

However, due to the openness of the communication environment and the complexity of the system structure, a MAS is very vulnerable to network attacks, which brings the risk of system instability. Fundamentally, a MAS benefits from the high efficiency of the system, but it is bound to lack a central integrated device to monitor and manage the activities of all nodes in the network. While high-intensity information exchange is required, it cannot verify the information flow in the system, making the system at risk of security problems. Therefore, it is necessary to study the resilient control structure of a MAS under fault and attack.

As for MASs, the cyber attacks that scholars have studied at present mainly include: denial of service (DoS) attacks [11], spoofing attacks [12], Byzantine attacks [13], replay attacks [14], covert attacks [15], actuator attacks [16], communication attacks [16], intelligent attacks [17], policy attacks [13] and so on. Among them, DoS and spoofing attacks are the two most typical and common network attacks in the field of MASs at present. In addition, the Byzantine attack has become an emerging and significant research topic in recent years. Thus, there are tremendous results concentrating on DoS attacks, spoofing attacks and Byzantine attacks. For this reason, this survey focuses on these three kinds of cyber attacks to illustrate the recent advances in these fields.

The motivations for the survey are twofold. Firstly, diverse control methods have been developed in the published literature throughout the last decade to explore the defense mechanisms for MASs under network attacks, which are not covered in the existing surveys [18,19,20,21,22]. Secondly, the related fields are mature enough to deserve a survey classifying the existing analytical approaches, the models used and the results achieved for the MASs under attacks from both systems and control perspectives. The contributions of this survey can be summarized as follows:

- It develops a comprehensive classification of resilient consensus strategies. The attack types discussed are more basic and comprehensive and can cover many special attacks developed from these three main attacks.
- It reviews an extensive set of more than 100 consensus algorithms and discerns the classes they are associated with. The attack mechanism and the corresponding security control protocol are described in terms of the formula definition and algorithm construct. The shortcomings of the control protocols are explained according to the specific parameters, so as to clearly analyze the corresponding security control framework.
- The algorithms in the same class are compared regarding their attack types, centralization, scalability and so on. This survey summarizes the main elastic control schemes corresponding to each attack as much as possible, which can be applied to a variety of actual scenarios and attack situations and provide solutions to security control problems.

The remainder of this paper is as follows. Section 2 presents the preliminary on graph theory and the consensus problem of MASs. Section 3 is dedicated to reviewing the work on DoS attacks, spoofing attacks and Byzantine attacks. Some challenging issues are raised in Section 4 to guide the future research.

## 2. Preliminaries and Consensus Problem for MASs

This section recalls some preliminaries about graph theory and some fundamentals on the consensus problem of MASs.

### 2.1. Preliminaries about Graph Theory

The information connection among agents can be modeled by a graph G(V,E), where V denotes the set of vertices $\left\{v_{1},\;v_{2},\;\ldots ,\;v_{N}\right\}$ which represents the set of agents in the system, and E⊆V×V gives the set of links which mimics the connection among agents. Given nodes *i* and j∈V, *i* can send information to *j* if there exists a directed edge from *i* to *j*, which is in the form of (j←i). The adjacency matrix associated with graph G is denoted by $A=[a_{ij}]$, where $a_{ij}=1$ if (j,i)∈E; 0, otherwise. G(V,E) is called an undirected graph if A is symmetric. The Laplacian matrix of G is defined by $L=[l_{ij}]$, where $l_{ij}=-a_{ij}$ if j≠i; $l_{ii}=\sum_{j=1}^{N}a_{ij}$ otherwise.

At time *t*, denote by G(t) the pair (V,E(t)), where the edge set E(t) varies with time. If there is a random process governing the change of G(t), one calls the communication graphs to be randomly switching.

### 2.2. Consensus Problem for MASs

The consensus control problem of MASs has attracted tremendous attention from researchers in the past decades. In general, suppose that the MAS consists of *N* agents with first-order integrator continuous-time dynamics described by

(1) $$\begin{array}{c} {\dot{x}}_{i}\left(t\right)=u_{i}\left(t\right),\;i=1,\ldots ,\;N, \end{array}$$

of which $x_{i}\left(t\right)\in R^{n}$ and $u_{i}\left(t\right)\in R^{n}$ denote, respectively, the state and control input of agent *i* at time *t*.

The consensus problem for agents described by (1) can be divided into the leaderless consensus problem and the leader-following consensus problem, according to the theoretical frameworks.

**Definition** **1.** *The leaderless consensus problem is to construct a controller for a MAS given by (1) such that*

(2) $$\lim_{t\to \infty }∥z_{i}\left(t\right)-z_{j}\left(t\right)∥=0,\;j\in N_{i},\;i=\;1,\ldots ,\;N,$$

*where $z_{i}\left(t\right)\in R^{m}$ and $z_{j}\left(t\right)\in R^{m}$ denote the state or output of agent i and j, respectively; $N_{i}$ represents the neighbor set of the i-th agent.*

**Definition** **2.** *The leader-following consensus problem is to construct a controller for a MAS given by (1) such that*

(3) $$\lim_{t\to \infty }∥z_{i}\left(t\right)-z_{0}\left(t\right)∥=0,\;i=\;1,\ldots ,\;N,$$

*where $z_{i}\left(t\right)\in R^{m}$ and $z_{0}\left(t\right)\in R^{m}$ denote the state or output of agent i and the leader, respectively.*

Moreover, the first-order discrete-time MAS is composed of *N* agents with dynamics given by

(4) $${\dot{x}}_{i}\left(k+1\right)=u_{i}\left(k\right),\;i=1,\ldots ,\;N,$$

of which $x_{i}\left(k\right)\in R^{n}$ and $u_{i}\left(k\right)\in R^{n}$ denote, respectively, the state and control input of agent *i* at time point k∈N. The above definitions can be correspondingly extended to the MAS given by (4).

Recently, the consensus problem for MAS with linearized or nonlinear dynamics have been studied in [23,24,25].

## 3. Resilient Control for MASs

This section reviews the results reported for the resilient consensus of MASs based on the limitations of the information interaction level and the constraints of the system level, respectively. Table 1 summarizes the classification of common cyber attacks of MASs. The information security problem of MASs is mainly carried out in three directions: information availability [26,27], confidentiality [28] and integrity [29]. It was gradually evolved into three specific research topics: attack detection, state observation and security control. Common network attacks mainly include denial of service attack that hinders information transmission [26,27,30,31], replay attack that repeatedly sends harmful information [32], deception attack (spoofing attack) that tampers with communication data [33], etc. From the limitations of information interaction, we focus on reviewing the following important and popular topics: the DoS attack, the spoofing attack and the Byzantine attack.

### 3.1. DoS Attack

**Figure 1:**
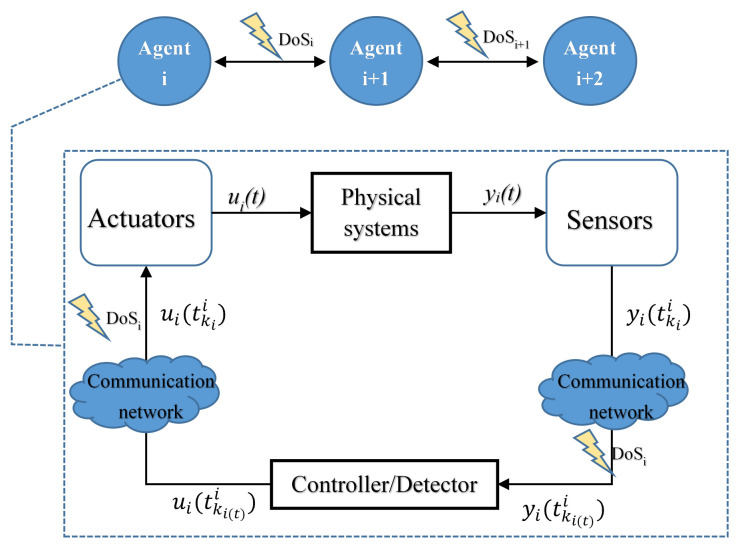
Multi-agent systems under DoS attack. DoS attacks may occur on the sensor–controller channel or the controller–actuator channel, affecting the transmission of system output signals and control signals, respectively. Hacker can also launch DoS attacks that can be independent of each other on different agents.

Network attacks can be divided into point attacks (agent dynamic behavior) and edge attacks (topology communication behavior) according to the attacked object. The former can be seen as the attacked agent being “moved out” of the topology [34], while the DoS attack belongs to the latter. That is, the control/measurement transmission channel is truncated by the attack, so that the target agent cannot obtain the signal, thereby damaging the availability of information, as shown in Figure 1. The DoS attack can be implemented by the attackers in several ways: filling buffers in a user or the kernal domain, blocking or jamming the communication among key components, and altering a routing protocol. Refs. [26,27,30,35], respectively, analyze the DoS attack in the form of centralized control and distributed control. Due to the excellent robustness of the latter, it has a broader theoretical exploration prospect than the former, but there is less relevant research at present. Researchers usually simulate the impact of the attack on the system through packet loss, serious delay or communication interruption. With the gradual progress of research, the hypothetical limitation of DoS attack model has been issued from the period known in advance [36] and gradually relaxed to the random occurrence mechanism of [30,31]. Generally speaking, the attack energy is always limited; that is, there is a period of energy accumulation time between adjacent attacks, which is called sleep time. During this period, communication can be carried out normally. If the sleep time is too short, the system will not be able to complete the transmission of control signals and the update of state values in time when the input information is lost for a long time, which may cause the irreparable loss of consensus.

In existing studies, in order to avoid the above extreme attacks, the attack frequency $F_{a}$ and attack duration $T_{a}$ of DoS attacks are generally limited according to the designed control structure parameters [30,31,32,33,37,38]. Their definitions in the time period $\left[T_{1},T_{2}\right)$ are as follows:

(5) $$F_{a}\left(T_{1},\;T_{2}\right)=\frac{N_{a}\left(T_{1},\;T_{2}\right)}{T_{2}-T_{1}},$$

(6) $$T_{a}\left(T_{1},\;T_{2}\right)\leq T_{0}+\frac{T_{2}-T_{1}}{\tau_{0}},$$

where $N_{a}$ denotes the attack number in the time period $\left[T_{1},T_{2}\right)$, $T_{2}\geq T_{1}\geq t_{0}$; $T_{0}>0$ and $\tau_{0}>1$ are scalars.

DoS attacks can paralyze the communication between agents, making the target agent unable to obtain the state information of the neighbor agents, thus, increasing the state error of the system. The trigger function of the event-triggered control mechanism is generally related to the state error, which can effectively judge the divergence of the system state error under the DoS attack, and can timely suppress the error divergence behavior. Therefore, the security control under DoS attack is mostly associated with the event-triggered mechanism [39]. While realizing flexible control, compared with periodic sampling control, it is more conducive to saving communication resources and avoiding network congestion. The event-triggered mechanism was proposed in [40,41] and then widely used in [31,42,43,44]. Event-triggered mechanism means that when the state deviates from the balance and exceeds the set threshold, the system stops feedback and triggers events to perform preset tasks, such as transferring information between neighbors or updating the controller. Compared with continuous-time control, an event-triggered mechanism requires additional consideration of the avoidance of Zeno behavior: that is, the situation of infinite triggering in an instant. This can be done by agreeing on the lower bound of the interval between two adjacent triggered instants [30,31] or introducing a bounded attenuation function into the event-triggered condition [44]. It can also be proved from the stability analysis that Zeno behavior does not exist [45].

According to the control structure, there are several schemes to achieve security consensus.

The simplest attack model is given in [31]; that is, the attack cycle and maximum duration are known in advance and constant, and a control scheme based on event-triggered mechanism under the leader-following topology is designed. It only allows events to be triggered during the communicable time period of the system. Under the condition that the attack parameters meet some system structural constraints, when the most serious imbalance occurs—that is, the time when the longest continuous attack ends—through the intervention of the control signal, the state quantity that deviates from the equilibrium can still be corrected, proving the feasibility of the elastic structure.

**Figure 2:**
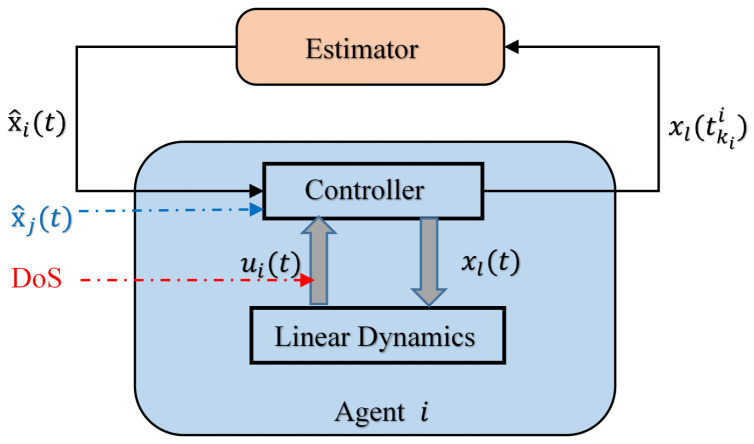
The event-triggered controller with an estimator under DoS attacks. ${\hat{x}}_{j}\left(t\right)$ and ${\hat{x}}_{i}\left(t\right)$ represent the estimates of agent *j* and agent *i* calculated by (7) and (8), respectively. $x_{j}\left(t_{k_{j}}^{j}\right)$ is the state value at the triggering time $t_{k_{i}}^{i}$, where $t_{k_{i}}^{i}$ is determined by the trigger function (11). Under the influence of DoS attack, (11) can be triggered by the system, so as to update the state estimated value according to (8) and adjust the control signal on the basis of (9).

In the attack environment of random mechanism, an open-loop observer is designed in [30], as shown in Figure 2. $t_{k_{i}}^{i}$ denotes the time series updated for the controller. $x_{l}$ and ${\hat{x}}_{i}$ represent the state quantity of the leader and the observed state quantity of followers, respectively. When the communication of the system is interrupted due to the attack, the observer is used to estimate the control signal and transmit it to the controller. The observer is as follows:

(7) $$\begin{array}{ccc} {\dot{\hat{x}}}_{j}\left(t\right) & = & A{\hat{x}}_{j}\left(t\right),\;t_{k_{j}}^{j}\leq t_{k_{j+1}}^{j}, \end{array}$$

(8) $$\begin{array}{ccc} {\hat{x}}_{j}\left(t_{k_{j}}^{j}\right) & = & x_{j}\left(t_{k_{j}}^{j}\right),\;j\in N_{i},\;k_{j}\in N, \end{array}$$

where the matrix *A* denotes the system matrix; N defines the set of integers. The control protocol for the multi-agent system is given by:

(9) $$u_{i}\left(t\right)=K{\hat{\xi }}_{i}\left(t\right),\;i=1,\ldots ,N,$$

where *K* is the control gain matrix, and

(10) $${\hat{\xi }}_{i}\left(t\right)=\sum_{j\in N_{i}\left(G\right)}a_{ij}\left({\hat{x}}_{j}\left(t\right)-{\hat{x}}_{i}\left(t\right)\right),\;i=1,\ldots ,N.$$

It follows from (10), that ${\hat{\xi }}_{i}\left(t\right)$ depends solely on the observed state, instead of the true state. The event-triggered condition is:

(11) $$∥e_{i}\left(t\right)∥\leq \beta_{i}∥{\hat{x}}_{i}\left(t\right)∥,\;0<\beta_{i}<1,\;i=1,\ldots ,N,$$

where $e_{i}\left(t\right)={\hat{x}}_{i}\left(t\right)-x_{i}\left(t\right)$ denotes the observed error. When MASs suffer from DoS attacks, the control protocol proposed by [46,47] may collapse because the control signal cannot be updated as expected (e.g., set to zero). Then, the system cannot reset the $e_{i}$ and gradually loses consensus. The above event-trigger strategy (11) and observer structure (7) and (8) can achieve resilient consensus under DoS attack. Setting the threshold depends on the state difference of the agent, which is the simplest form of the state-dependent triggered mechanism. The parameter $\beta_{i}$ needs to be selected from the compromise between the performance measurement of system convergence speed and communication frequency.

Since this estimate is designed based on the model, it means that the system can be unstable. The update process of the system can be summarized as follows: the observer calculates and estimates the state value of the neighbor according to the dynamic equation until the next triggered time $t_{k_{j}}^{j}$ comes. ${\hat{x}}_{j}\left(t\right)$ is updated to the state value passed by the neighbor at this time, and its own state value $x_{i}\left(t\right)$ is read and updated at the same time. The significance of signal separation is that if continuous communication cannot be achieved, it will not affect the triggering of events. When the system is attacked by DoS, the control signal will not be set to zero for a long time due to the failure to reset $e_{i}\left(t\right)$, thereby losing consensus [40,47].

The scheme in [40,47] can achieve exponential consensus in the two cases of leaderless and leader-following under DoS attacks, but the corresponding topology requirements are different: the former requires that the topology graph should be connected, while the latter requires that a directed spanning tree should be included. At the same time, both schemes can only be achieved when the attack frequency and duration have upper bounds. Additionally, both control schemes are not completely distributed. They use global information, namely the eigenvalues of the Laplace matrix, to design the matrix parameters of the controller, which is not conducive to the application of large-scale agent systems. The core of this scheme is to solve the problem of control interruption caused by failure to communicate by observing the state of neighbors. In addition, additional error variables $e_{i}^{\mathrm{dos}}=x_{i}\left(t\right)-x_{i}\left(t_{m}\right)$ can be introduced into the triggering function, where $t_{m}$ is the starting time of the attack, which can effectively reduce the number of unnecessary triggers of events [44].

Different from [30] and using the state measurement error $e_{i}\left(t\right)$ as the triggered condition, ref. [38] designs a control scheme based on the topology input triggered mechanism and gives the corresponding state observer: Given i,j=i=1,…,N, and i≠j, define the state corresponding to edge (i,j) as

(12) $$\omega_{ij}\left(t\right)≜x_{i}\left(t\right)-x_{j}\left(t\right),$$

which satisfies

(13) $${\dot{\omega }}_{ij}\left(t\right)=A\omega_{ij}\left(t\right)+B\left(u_{i}\left(t\right)-u_{j}\left(t\right)\right),$$

where *B* is the input matrix.

After the control is triggered, the variables $\omega_{ij}$ cannot be measured until the next triggered time comes. Considering this fact, use an observer for estimation:

(14) $${\dot{\hat{\omega }}}_{ij}\left(t\right)=A{\hat{\omega }}_{ij}\left(t\right).$$

When the triggered time of the sleep time comes, assign the value directly as follows:

(15) $${\hat{\omega }}_{ij}\left(t_{ijk_{i}}^{i}\right)=\omega_{ij}\left(t_{ijk_{i}}^{i}\right).$$

Define topology state prediction error as:

(16) $${\tilde{\omega }}_{ij}\left(t\right)={\hat{\omega }}_{ij}\left(t\right)-\omega_{ij}\left(t\right),\;t\in \left[t_{ijk_{i}}^{i},t_{ijk_{i}}^{i+1}\right).$$

When the triggered time $t_{ijk_{i}}^{i}$ comes, the real value $\omega_{ij}$ is assigned to the estimated value ${\hat{\omega }}_{ij}$. Before the time $t_{ijk_{i+1}}^{i}$ comes, the estimated value is provided as an input to the controller for adjustment:

(17) $$u_{i}\left(t\right)=-\alpha K\sum_{j\in N_{i}}{\hat{\omega }}_{ij}\left(t\right),$$

where the scalar α denotes the coupling gain. The triggered instant and the triggering function corresponding to edge (i,j) is

(18) $$\begin{array}{ccc} t_{ijk_{i+1}}^{i} & = & \inf\{t|t>t_{ijk_{i}}^{i},f_{ij}\left(x_{i}\left(t\right),u_{i}\left(t\right),t\right)>0\mathrm{or}f_{ji}\left(x_{i}\left(t\right),u_{i}\left(t\right),t\right)>0\}, \end{array}$$

where

(19) $$\begin{array}{ccc} f_{ij}\left(x_{i}\left(t\right),u_{i}\left(t\right),t\right) & = & ∥\int_{t_{ijk_{i}}^{i}}^{t}e^{A(t-s)}Bu_{i}{\left(s\right)ds∥}^{2}-\beta_{ij}∥B^{T}Qx_{i}{\left(t\right)∥}^{2}{∥B^{T}Q∥}^{-2}>0, \end{array}$$

(20) $$\begin{array}{ccc} f_{ji}\left(x_{j}\left(t\right),u_{j}\left(t\right),t\right) & = & ∥\int_{t_{ijk_{j}}^{j}}^{t}e^{A(t-s)}Bu_{j}{\left(s\right)ds∥}^{2}-\beta_{ji}∥B^{T}Qx_{j}{\left(t\right)∥}^{2}{∥B^{T}Q∥}^{-2}>0,\\ & & t\in [t_{ijk_{i}}^{i},t_{ijk_{i}}^{i+1}), \end{array}$$

in which $Q\in R^{n\times n}$ is a positive definite matrix.

When the topological edge is attacked by DoS, $∥{\tilde{\omega }}_{ij}\left(t\right)∥$ will break through the normal upper bound and the above triggered conditions will be met. $\beta_{ij}$ is the parameter of triggering function corresponding to the edge (i,j). Thus, the triggering threshold and the lower bound of adjacent triggered time can be flexibly set according to different edges, which reflects the distributed characteristics of event-triggered control. $f_{ij}$ just depends on $u_{i}$ and $x_{i}$, which are the state values of an agent itself. Unlike [30], which uses the neighbor’s state difference as the triggered condition, ref. [38] does not require each agent to continuously broadcast its own control input and state quantity, which well maintains their own information privacy. In addition, the communication and the update of the state value can only be carried out when the state of the topological edge meets the triggering function, compared with the continuous information transmission and reception required in [30] to calculate the state observation value of the neighbor and its own real state value. Thus, the former obviously reduces the communication cost and calculation complexity, but the corresponding parameter design is much more difficult. Moreover, compared to the general state consensus realized in [30], ref. [38] can achieve more accurate consensus results as exponential consensus.

Based on the information of the Laplace matrix eigenvalues of the communication topology, the control scheme designs the trigger parameter $\beta_{ij}$ in the normal communication scenario, and then, achieves the state consensus of the system. In the case that the communication situation is under DoS attack, it can be shown that when the DoS attack frequency and the attack duration satisfy the inequality condition (related to the sleep time of DoS attack), the controller (17) can mitigate the communication anomaly caused by the DoS attack and achieve the security consensus of the system. Meanwhile, the design of parameters in both the controller (17) and the trigger function (18) relies on global information such as the non-zero minimum eigenvalue of the Laplace matrix. Thus, it is not a fully distributed control structure.

Considering the periodic DoS attack, ref. [48] models the error dynamics of the MASs as a switched time-varying delay system and proposes an event-triggered mechanism control protocol using the input time delay method, finally achieving the exponential consensus of the system. Its highlight is that, based on the existing theoretical research of time delay MASs, it converts the sampled-data term into a time delay term in the system. In addition, according to the sleep time of different DoS attacks, ref. [48] also puts forward an optimization algorithm to select the control parameters of the distributed event-triggered protocol. However, the attack is required to occur periodically. Meanwhile, the exact value of attack cycles and sleep time need to be known in advance, which makes [48] less applicable.

To sum up, for the resilient consensus problem under DoS attack, the current control scheme with high feasibility is to use the observer to simulate the evolution of normal state values during the communication paralysis period and combine the asynchronous triggered scheme to achieve consensus under the premise of limiting the attack frequency and duration. According to the accuracy and complexity of the observer and the sensitivity of the triggered mechanism, the conservatism and practicality of the corresponding consensus conclusions are different. To improve the practicability of the conclusion, the key lies in whether the attack model can fully ensure the accuracy of the prediction of the attacked object and the reduction of the actual damage.

In order to give a clear survey, we summarize the relevant work in Table 2 according to DoS type, centralized, scalability, results and references.

Ref. [47] studies the problem of fault detection and consensus control under DoS attack and proposes an attack model based on the hidden semi Markov process for the first time. It can effectively meet the conditions of stealth of attack strategy and complexity of behavior. When a DoS attack comes, the zero-order holder will maintain the amplitude of the last normal input signal. At this time, the system will change from the original uniform sampling system to the non-uniform sampling system, and the sampling period will also change. Using this property, we can define a working mode set $\phi {\left(k\right)}_{k\in N}\in R≜\left\{1,\;2,\;\ldots ,\;R\right\}$ when the system is attacked, so that we can obtain the emission probability of the system by using a semi Markov kernel and probability density function of mode-dependent dwell time τ only through sample data:

(21) $$\Psi \left(\phi \left(k\right),\eta_{k}^{\delta_{k}}\right)≜\mathrm{Pr}\left\{\eta_{k}^{\delta_{k}}|\phi \left(k\right)\right\},$$

where $\eta_{k}^{\delta_{k}}\in M$ denotes the set of attack models observed by the system, and $\delta_{k}\leq \tau$ defines the running time of current mode. Let $l(\delta_{k})$ be the $\delta_{k}$-th observer mode, when the real attack mode is a∈R. Through hidden Markov theory [50], a finite set of observation patterns ${\hat{M}}_{a}$ can be obtained:

(22) $$\begin{array}{ccc} {\hat{M}}_{a} & = & \left\{0<\Phi \left\{a,l\left(\delta_{k}\right)\right\}<1;\;l\left(\delta_{k}\right)\in M;\;1\leq \delta_{k}\leq \tau \right\}\\ & = & \{{\hat{l}}_{\delta_{k}}^{1}\left(a\right),\;{\hat{l}}_{\delta_{k}}^{2}\left(a\right),\ldots ,{\hat{l}}_{\delta_{k}}^{g}\left(a\right)\}, \end{array}$$

where $g≜|{\hat{M}}_{a}|$, ${⋃}_{a=1}^{R}{\hat{M}}_{a}=M$ and

(23) $$\sum_{l_{\delta_{k}}={\hat{l}}_{\delta_{k}}^{1}\left(a\right)}^{{\hat{l}}_{\delta_{k}}^{g}\left(a\right)}\Phi \left\{a,l\left(\delta_{k}\right)\right\}=1.$$

It follows from (23) that in the observation pattern set, there will always be patterns that match the real attack situation. According to the observation mode obtained by the system, the detection and control structure solely depend on the sampled data without knowing the attack statistics.

In addition, ref. [43] discusses the secure synchronization of MAS with linear dynamics under DoS attack with Markov model, but the communication resources are required to be infinite. Refs. [50,51] study the robust tracking problem when the system encounters two kinds of network attacks: communication hold and communication interruption. The switching state of the system under intermittent communication is described by the stochastic Markov process, and the control goal is achieved by using the periodic sampling control scheme based on time series. Ref. [44] discusses the situation that different topological edges of the system are attacked by different DoS at the same time: according to the number of attacked edges, it defines different limiting conditions for the attack and different triggering conditions for the controller. However, it essentially designs different schemes for all subsets of topological edges that are attacked by the same kind of DoS at the same time. Recent studies have also shown a new trend that combines DoS attacks with other system constraints such as failures, interference, saturation, etc. For example, ref. [37] combines DoS attacks with input saturation. On the premise that the system can be stabilized, it uses the small gain theory to linearize, realizing the semi-global security consensus of the system. Ref. [52] designs an observer to solve the synchronization problem of a discrete MAS when the lossy sensor with state threshold exists simultaneously with the network attack. According to whether the communication topology is directed or not, whether the leader exists or not, as well as the system order and isomorphism, the attack model will also be adjusted accordingly, and different theoretical results will be obtained: for example, ref. [10] studies the exponential consensus problem under the directed topology, and [45,53] achieve the ultimate bounded consensus under the undirected topology. Ref. [54] explores a robust output consensus scheme for heterogeneous multi-agent systems under random DoS attacks. Refs. [55,56] design a control scheme based on self triggering, bypassing the disadvantage that event triggering requires continuous monitoring. As long as the current state value of the agent is known, the next triggering time can be calculated. In the case of maximizing the limitation of attack frequency and duration, a pulse controller with observer structure is proposed in [49] and a state reset method based on measurement error is given. In [57], considering the saturation of the system state and the gain disturbance of the control protocol caused by the network attack and communication congestion of the MAS, combined with the polling method, a robust optimal controller is given to achieve the security consensus of the MAS in the infinite time domain. Ref. [58] points out the challenges that network security brings to MAS in terms of autonomy and information interdependence. They provide a set of basic principles of network security science. The detection algorithm and mitigation algorithm of MAS for network attack are studied in [59], and a practical application example is given based on the distribution automation system. We addressed the robust secure consensus problem in [3] and the antiwindup secure control consensus issue in [35]. It is worth mentioning that the secure consensus protocols proposed in [3,35] are fully distributed secure consensus protocols without using any global information of the communication topologies.

### 3.2. Spoofing Attack

A MAS can be regarded as a special cyber-physical system. Its controller not only needs to obtain data from sensors, but also needs to send control information to actuators. Network attacks may occur on the channels from a sensor to a controller and from a controller to a actuator—even the controller itself may be attacked. For attackers, in addition to interrupting the information loop, tampering with the data being used for communication can also achieve destructive effects, which is a deception attack.

A spoofing attack refers to an attack in which an attacker can obtain system information and perform arbitrary operations on measurement data and control instructions. It can bypass the detection device and make the agent adopt an error value. Common false data injection (FDI) [60] attacks also fall into the scope of spoofing attacks, as well as data replay [61], data change [12] and other types of attacks. Compared with DoS attacks, deception attacks are more difficult to detect. They exist in power networks [62], smart grids [63] and other fields, which greatly threaten the integrity of data [64].

Refs. [64,65], respectively, consider the spoofing attack on the sensor–controller channel and the controller–actuator channel. Ref. [66] uses the indicator vector to model the deception attack and discusses the situation that the attack occurs on the sensor and actuator at the same time. Generally speaking, the establishment of attack model always revolves around the change of data. Similar to DoS attacks, the research of deception attacks is generally carried out in discrete-time systems, and the control methods adopted include periodic control, event-triggered control, pulse control, etc. Among them, pulse control is more commonly used because it allows discontinuous input and has the characteristics of instantaneous jump, which is very appropriate to deal with some deceptive attacks that implement state mutation.

**Figure 3:**
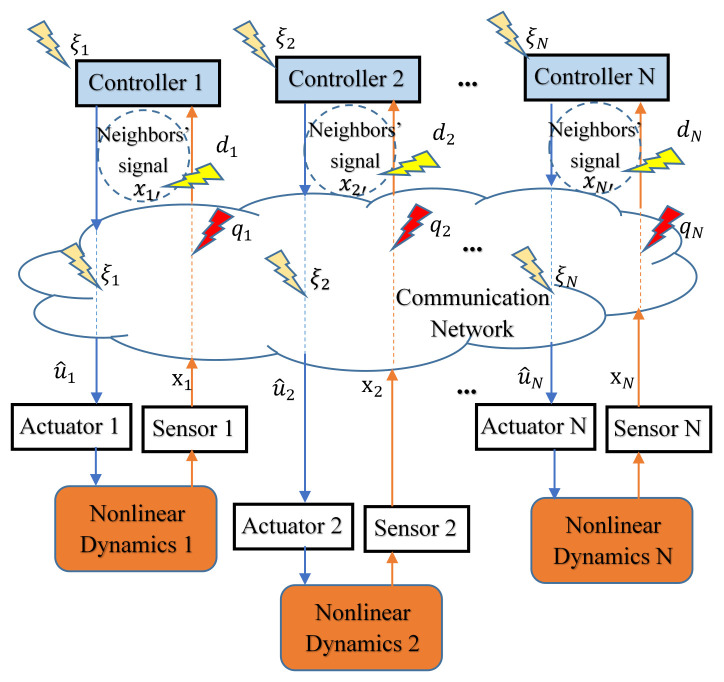
The deception attack in cyber-physical system. According to the secific location of the deception attack, the figure shows three different deception attacks, where $\xi_{i}$ represents the deception attack on the controller itself or controller–actuator channel (tampering with the control signal $u_{i}$), $q_{i}$ represents the deception attack on the sensor–controller channel (tampering with the output signal $y_{i}$), and $d_{i}$ represents the deception attack on the transmission channel of the neighbor agents (tampering with the state value $x_{j}$ with $j\in N_{i}$).

In [67], for a Lipschitz-type nonlinear MAS under deception attack, a mean-square bounded synchronization control scheme based on distributed impulse control is proposed. Given i=1,…,N, the Bernoulli distribution with parameter β is used to model the displacement type spoofing attack behavior on the controller–actuator channel, as shown in Figure 3.

When the attack succeeds, replace the control input signal $u_{i}$ with an error signal $\psi_{i}$:

(24) $$u_{i}\left(t\right)=c\sum_{k=1}^{\infty }\left(-\sum_{j=1}^{N}l_{ij}x_{j}\left(t\right)-d_{i}\left(x_{i}\left(t\right)-x_{l}\left(t\right)\right)\right)\delta \left(t-t_{k}\right),$$

(25) $${\hat{u}}_{i}\left(t\right)=\sum_{k=1}^{\infty }\left[\beta_{i}\left(t\right)\psi_{i}\left(t\right)+\left(1-\beta_{i}\left(t\right)\right)u_{i}\left(t\right)\right]\delta \left(t-t_{k}\right),$$

where $\mathrm{Prob}\left\{\beta_{i}\left(t\right)=1\right\}=\bar{\beta }$; $\mathrm{Prob}\left\{\beta_{i}\left(t\right)=0\right\}=1-\bar{\beta }$; δ denotes the Dirac pulse; $d_{i}$ is the corresponding element value of the *i*-th degree matrix; *c* is the coupling strength, and it is assumed that $\beta_{i}$ is independent and the attack signal is bounded: $∥\psi \left(t_{k}\right){∥}^{2}\leq \psi$.

For the MASs with the following nonlinear dynamics:

(26) $${\dot{x}}_{i}\left(t\right)=Ax_{i}\left(t\right)+Bf\left(x_{i}\left(t\right)+{\hat{u}}_{i}\left(t\right)\right),\;i=1,\ldots ,N,$$

(27) $${\dot{x}}_{l}\left(t\right)=Ax_{l}\left(t\right)+Bf\left(x_{l}\left(t\right)\right),$$

where $x_{i}$ is the state of the *i*-th follower, $x_{l}$ is the state of the leader and f(·) is a Lipschitz type nonlinear function.

The protocol defines a parameter μ, which corresponds to the Laplace matrix, degree matrix, coupling strength and attack strength. By limiting the value range of μ, the communication topology can be appropriately designed according to the attack situation. At the same time, when μ satisfies the linear matrix inequality condition of the pulse interval parameter $h_{2}$—that is, when the pulse interval as well as the topological coupling coefficient match the strength of the deception attack and the attack probability—the control protocols (24) and (25) can successfully defend the deception attack and the followers (26) track the leader (27) in the mean square sense. The selection of coupling strength *c* and pulse interval parameter $h_{2}$ are related to the global information of communication topology, so the control protocol is not completely distributed. In addition, the system dynamics contains random variables about deception attack as $\beta_{i}$. Thus, it can only realize the target of bounded synchronous tracking in the mean square sense. Compared with the case of no attack, $\bar{\beta }$ increases the upper bound of the synchronization error and narrows the pulse spacing, which also puts forward corresponding requirements for *c*. That is why $\bar{\beta }$ is always restricted.

Compared with [67], ref. [68] studies the overlay spoofing attack on the sensor–controller channel, as shown in Figure 3. The attack causes the normal state value to be superimposed with the error value $q_{i}\left(t\right)$:

(28) $$u_{i}\left(t\right)=\sum_{k=1}^{\infty }\left[c\sum_{j=1}^{N}\left[-l_{ij}x_{j}\left(t\right)+\beta_{ij}q_{i}\left(t\right)\right]-cd_{i}\left(t\right)\left[x_{i}\left(t\right)-x_{l}\left(t\right)\right]\right]\delta \left(t-t_{k}\right).$$

Ref. [68] also achieves mean square bounded synchronization, but only the size of the upper bound of the error is affected by $\bar{\beta }$. Regardless of the attack mode, the synchronization performance is mainly affected by the coupling strength, degree matrix, attack probability and pulse spacing. In particular, the first two terms play a decisive role in reducing synchronization error when the probability of each side being attacked is the same.

Most of the above research fail to effectively eliminate the impact of spoofing attacks on MASs, resulting in the boundedness of their final consensus. As for that, ref. [69] proposes a distributed filter with adaptive compensator as follows:

(29) $$\left\{\begin{array}{c} {\dot{\psi }}_{i}\left(t\right)=E\psi_{i}\left(t\right)+{\hat{\varepsilon }}_{i}\left(t\right)\\ {\hat{\varepsilon }}_{i}\left(t\right)=-{\hat{\varepsilon }}_{i}\left(t\right)+\varepsilon_{q,i}^{a}\left(t\right)+\rho_{a,i}\left(t\right)\\ \rho_{a,ij}\left(t\right)=-\frac{F_{i}^{T}{\hat{\varepsilon }}_{i}\left(t\right){\hat{\delta }}_{a,ij}^{2}\left(t\right)}{∥F_{i}^{T}{\hat{\varepsilon }}_{i}\left(t\right)∥{\hat{\delta }}_{a,ij}\left(t\right)+\vartheta_{ij}\left(t\right)} \end{array}\right.$$

where $\psi_{i}\left(t\right)$ and ${\hat{\varepsilon }}_{i}\left(t\right)$ are the states of the observer and filter, respectively. $\varepsilon_{q,i}^{a}\left(t\right)$ is the relative observation, and $\rho_{a,i}\left(t\right)=\sum_{j\in N_{i}}a_{ij}\rho_{a,ij}\left(t\right)$ is the compensation term. ${\hat{\delta }}_{a,ij}\left(t\right)=-\lambda \sigma_{ij}\left(t\right){\hat{\delta }}_{a,ij}\left(t\right)+\lambda ∥F_{i}^{T}{\hat{\varepsilon }}_{i}\left(t\right)∥$, $\sigma_{ij}\left(t\right)$ is a bounded signal. ${\hat{\delta }}_{a,ij}\left(t\right)$ is the estimate of the spoofing attack signal.

Under the function of the filter, the system can compensate the offset of consensus error variables caused by spoofing attacks and achieve accurate consensus result without error. The distributed controller only uses the local information of the agent itself and does not need to use the random characteristic and upper bound of the attack signal, which is more conducive to distributed applications. However, the design complexity of the controller is related to the dimension of the system state, which makes it extremely difficult to design its control parameters for complex systems.

The assumption that the attack is bounded and obeys Bernoulli distribution made in [67,68] is very conservative. It also stipulates that the error values received by the attacked agents are the same and will not vary as the topology changes, resulting in low practicability of the conclusion. Ref. [70] studies the substitution spoofing attack on the transmission channel of the neighbor node, as shown in Figure 3. It only borrows the concept of the ”F-local” to limit the maximum number of simultaneous attacks:

(30) $$|\;\{j\in N_{i}\left(k\right):\left(j,i\right)\in E_{A}\left(k\right)\}|\leq F,\;F\in R,$$

where $E_{A}\left(k\right)$ is the topological edge set that is attacked at time point *k*. Let $E_{T}\left(k\right)$ be the trust edge set that is known to not be attacked:

(31) $$x_{j}^{i}\left(k\right)=d_{j}^{i}\left(k\right),\;j\in N_{i},\;\left(j,i\right)\in E_{A}\left(k\right),$$

(32) $$x_{j}^{i}\left(k\right)\equiv x_{j}\left(k\right),\;j\in N_{i},\;\left(j,i\right)\in E_{T}\left(k\right),$$

where $x_{j}^{i}\left(k\right)$ denotes the information received by node *i* through the edge (j,i) at time point *k*, and $d_{j}^{i}\left(k\right)$ represents the error value.

It can be proved that as long as the agent has a trust entry edge, all of its state values will always fall under a trust state value on the interval. Suppose that H(k) and h(k), respectively, represent the largest and minimum state values. When the deception attack occurs on the entry edge of agent *i*, one has $x_{ij}\notin \left[h\left(k\right),H\left(k\right)\right]$. If $x_{ij}$ is used to update the controller, the agent *i* may output an abnormal state value, which can lead to the extension of adjustment time, or even system instability. Then, one can design a weighted average sub-sequence reduction algorithm with trust edge set based on [71], so as to complete the screening of problematic state values. The algorithm can be summarized as the following: when there is no trust edge, from the sequence of state values sorted according to size obtained at each time, taking its own state value as the boundary, the first *F* and the last *F* state values are screened out; However, if there are trust edges, only the trust values will be sorted and directly used for updating, and other state values will not be processed:

(33) $$x_{i}\left(k+1\right)=x_{i}\left(k\right)+c\sum_{j\in R_{i}\left(k\right)}a_{ij}\left(k\right)\left(x_{j}^{i}\left(k\right)-x_{i}\left(k\right)\right),\;i=1,\ldots ,N,$$

where $R_{i}\left(k\right)$ is the set of state values for updating after screening. If updated according to the above method, $x_{i}\left(k+1\right)\in \left[h\left(k\right),H\left(k\right)\right]$ will always be satisfied and extreme deviation due to attack will not occur. When there is no trust edge set in the system, if the topology has 2F+1 robustness [71], the system can achieve elastic consensus under *F*-local deception attacks. Compared with [67,68], (32) does not introduce random variables of spoofing attacks, so it can achieve more accurate consensus. When the system topology is time-varying G(k)=(V,E(k)), the robustness condition needs to be satisfied in a sufficiently long continuous time series to ensure that each node is sufficient to screen the sequence and broadcast the state values accurately. When the system has a trust edge set, the situation is similar, except that the condition of robustness should be consistent with the trust edge set: that is, it should have 2F+1 generalized robustness with respect to $E_{T}$ [70]. In order to further improve the practicability of the conclusion, ref. [70] also considers the situation that the topology does not meet the robustness. If the topology G(k)=(V,E(k)) has a spanning tree in a sufficiently long continuous time—that is, the consensus topology has a spanning tree—it can also achieve elastic consensus in a time-varying topology. Further, when $G_{T}\left(k\right)=\left(V,E_{T}\left(k\right)\right)$ contains a spanning tree, the goal can also be achieved. When the *F*-local hypothesis is no longer satisfied—that is, when the number of attacks is uncertain—as long as $G_{T}\left(k\right)=\left(V,E_{T}\left(k\right)\right)$ has a spanning tree and the root node only has a trust entry edge, consensus can also be achieved.

Since spoofing attacks involve the operation of communication data, they are generally more difficult to deal with than DoS attacks that truncate channels. On the one hand, according to the constraint conditions of the attack, the transmission frequency of the control signal and the coupling strength of the communication topology can be designed, and the distributed pulse control can be used to achieve the certain state synchronization. However, the restrictions on the attack situation are generally strong and conservative; On the other hand, a filtering algorithm can be designed to exclude data values suspected of tampering. After this, a secure state update can be carried out, but the corresponding topology needs to be specially constructed. At the same time, since the algorithm can only screen out problematic extreme values, it may be difficult to deal with more complex attacks.

**Figure 4:**
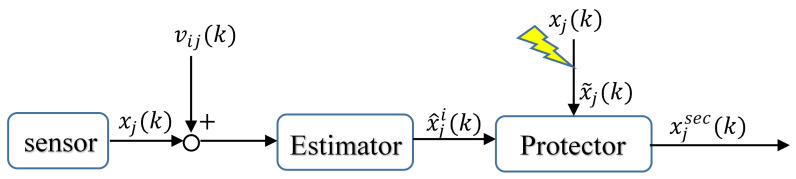
The schematic diagram of the screening mechanism. $x_{j}\left(k\right)$ represents the state value of agent *j* obtained by the sensor, and $v_{ij}\left(k\right)$ is the sensor noise in agent *i*. ${\hat{x}}_{j}^{i}\left(k\right)$ is the state value estimated by agent *i*, and ${\tilde{x}}_{j}\left(k\right)$ is the state value that may be tampered by the deception attack. Through the screening mechanism of the protector, the system can finally obtain the secure state value $x_{j}^{sec}\left(k\right)$.

In addition, there are also methods performing security control through state observation [72]. Ref. [60] establishes an observer based on the Kalman filter and realizes the system mean square consensus under the influence of Gaussian white noise and FDI attack. Different from the former, while observing, it also proposes a threshold comparison scheme to decide whether to use the observed value as the input of the next time step, as shown in Figure 4:

When the difference between the actual value of the state ${\tilde{x}}_{j}\left(k\right)$ and the observed value ${\hat{x}}_{j}^{i}\left(k\right)$ does not meet the preset threshold, observation will be enabled for control. That is,

(34) $$x_{i}\left(k+1\right)=x_{i}\left(k\right)-\epsilon \sum_{j=0}^{N}ll_{ij}\left(k\right)\left[\theta_{ij}\left(k\right){\tilde{x}}_{j}\left(k\right)+\left(1-\theta_{ij}\left(k\right)\right){\hat{x}}_{j}^{i}\left(k\right)\right)]+\omega_{i}\left(k\right),$$

(35) $$\theta_{ij}\left(k\right)=\left\{\begin{array}{cc} \; & 1,\;\mathrm{if}∥{\tilde{x}}_{j}\left(k\right)-{\hat{x}}_{j}^{i}(k)∥\leq \phi \left(k\right)\\ \; & 0,\;\mathrm{otherwise}. \end{array}\right.$$

However, the observer embedded in each agent contains the information of system dynamics structure and communication topology. The control structure is thus not completely distributed.

Ref. [73] studies the detection and identification of physical faults and FDI attacks and designs an exception handling mechanism with independent detector and cooperative detector through a $H_{\infty }$ multi-objective optimization method. The former is used to judge whether faults and attacks exist, and the latter is used to distinguish the two kinds of anomalies. At the same time, it puts forward the concept of intermediary centrality, which well describes the possibility of different topological edges being attacked so that detection resources can be reasonably allocated. Then, sensitivity and accuracy can be improved. However, there is a compromise between the accuracy and the robustness of the system to disturbances.

Ref. [74] obtains the necessary and sufficient conditions for the system under FDI attack to lose security consensus, according to which the prediction error can be set to an arbitrary value by bypassing the detection mechanism. Ref. [75] constructs a distributed filter against spoofing attacks by using a network composed of sensors of itself and of neighbors. Starting from the characteristic that both belong to opposite side attacks, ref. [76] studies the discrete-time stochastic system under DOS attack and deception attack and achieves consensus through event-triggered control. Ref. [77] gives a detection structure based on a neural network to judge whether the system is attacked by FDI. Ref. [78] proposes the concept of “competitive interaction” to design a flexible and cooperative control mechanism to achieve consensus under FDI attacks.

In order to give a clear survey, we summarize the recent work in Table 3 according to methodologies, attack location, spoofing attack type, centralized, scalability and references.

### 3.3. Byzantine Attack

Imagine a group of unmanned aerial vehicles (UAVs). If the attacker knows several UAVs in the group in advance and continuously sends wrong navigation data to nearby aircraft groups through them, it is obvious that this will cause the group to deviate from the pre-orientation as a whole. It can even cause the group to crash due to the lack of coordination between the air frames. We call these UAVs that continuously launch attacks “abnormal agents”. We call the problem that the system must achieve consensus to a variety of state variables in this environment “elastic consensus”.

Abnormal agents are generally divided into two types: fault agents and malicious agents. The former is caused by changes in the environment, without human subjective factors, and may cause abnormal updates of the agent’s state; The latter is a malicious agent designed by human beings to damage the testability of the system, which may block the operation of the system.

Byzantine attacks describe the attack situation when there are malicious agents in the agent network. Specifically, a malicious agent is an agent that satisfies one of the following three conditions:

1. The state value will not be updated as set.
2. Do not transmit its real state value to at least one outgoing neighbor.
3. The state values transmitted to different outgoing neighbors at the same time are inconsistent.

**Figure 5:**
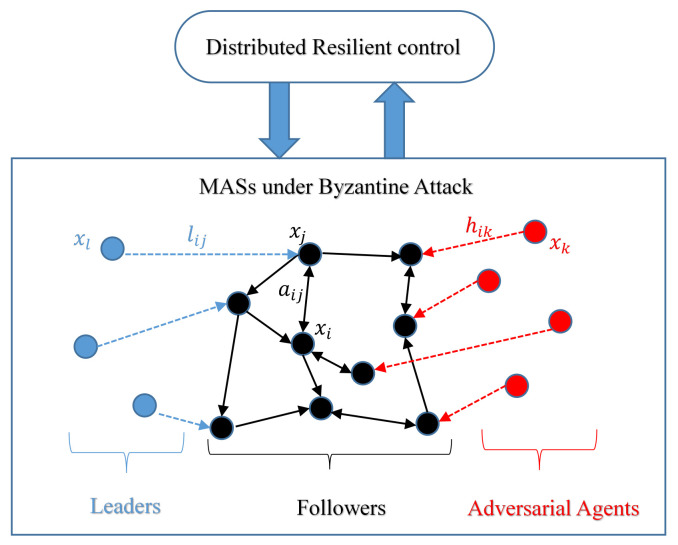
The diagram of a Byzantine attack. When the communication topology of agent *i* meets certain conditions, each follower $x_{i}$ can not only directly receive the data of neighbor agent $x_{j}$ ($a_{ij}\neq 0$), but also directly receive data from leaders $x_{l}$ ($l_{ij}\neq 0$) and adversarial agents $x_{k}$ ($h_{ik}\neq 0$). It is worth noting that the abnormal state information sent by $x_{k}$ may lead the system to an unexpected direction or even cause the system to crash.

The focus of their attacks is to send malicious state values to normal agents to make the system disordered. That is, the agent group mistakenly regards the malicious agent as the leader of the system, so that the system state value is guided to a harmful range, as shown in Figure 5. The malicious state value refers to a value that does not conform to the change direction of the normal state value of the system. The key to the problem is to screen out the malicious state value or shield its impact to the maximum extent.

Different from the error state value of the above substitution type spoofing attack, the malicious state value of the Byzantine attack is sent by an agent that originally exists in the system topology, while the former is sent by an external attacker. In terms of detection and security control, there will be differences between the two.

To achieve security consensus under the Byzantine attack, there are three main solutions: scheme one is to detect and screen malicious agents in advance, scheme two is to set up trusted decision-making agents, and scheme three is to propose an elastic control structure to maintain the system state or output within a tolerable range. Among them, scheme one is based on the robust structure of the graph, while scheme two generally sets up “trust nodes” to relax the robustness conditions of the graph. The two principles are similar. If the research object of scheme three is a heterogeneous system, it needs to use adaptive control structure or output feedback control structure to adjust under the premise of considering sensor error. The introduction of the concept of topology robustness gradually extends the deployment conditions of attack coping strategies to the communication structure, which can be achieved by modifying on the original topology [79] or constructing a special k-cycle graph [80].

Most of the existing research refers to the mean subsequence reduction (MSR) algorithm under the branch of distributed fault-tolerant control algorithm in the computer field to screen out the state values. However, it can only screen out the state variables with extreme values and has no ability to detect abnormal state values. In order to ensure that the screened state sequence still retains enough state quantity to maintain normal operation, the MSR algorithm generally needs to be applied in combination with graph robustness theory. At the same time, the concept of “*F*-total” and “*F*-local” should be used to limit the maximum number of malicious agents in the system [71]. In fact, the *F*-local type Byzantine model can be transformed into the above *F*-local type deception attack model as long as the outgoing edge of the Byzantine node is regarded as suffering from a substitution type deception attack. Otherwise, it is not true.

In the absence of external input, the final state value of the consensus achieved by this method always falls in a convex hull composed of the initial state value of the normal agents [81]; this final value will be affected by the behavior of the malicious agent. For the leadership system, the ideal situation is to track the leader’s state reference value by followers while shielding the impact of attacks. However, since the reference value may not fall into the above convex hull, further improvement is required.

Ref. [82] uses the modified sliding window MSR algorithm to solve the problem of elastic inclusion control in multi-leader systems and successfully makes followers track leaders whose state values are outside the convex hull. The parameter *F* in the algorithm is the corresponding parameter *F* in the *F*-local model to limit the maximum number of malicious agents. The time window loosens the robustness requirements of the topology map and no longer requires robustness to be maintained at every time step. It can be proved that, under the condition that graph G(T) has strong $\left(T,t_{0},2F+1\right)$ robustness to the leader set L, the system can track the leader state values outside the upper convex hull. This is because the robustness of the topology ensures that there is at least one state value of a normal leader in the filtered state value sequence. Since the design of MSR algorithm needs to obtain the maximum number *F* of malicious agents in advance, the control protocol is not completely distributed. At the same time, the controller puts forward robustness requirements for communication topology, which makes the controller unsuitable for large-scale MASs.

In the context of the security consensus of MAS on network attacks, in [83] for Byzantine attacks, two improved schemes based on MSR algorithm which relax the strict restrictions of the algorithm on network topology, improve the convergence speed of the system and solve the compatibility problem with clock synchronization are proposed.

Ref. [82] proposes using the sliding window approach to transform the elastic consensus problem in the continuous domain into the discrete domain, making it possible to further improve the scheme through the event-triggered mechanism [84]. The main difficulty in applying event-triggered control to MSR is the processing of the difference between the current state value and the last passed state value. Ref. [84] regards the difference of the above state variables as non-attenuated noise and uses it as the basis for triggering. They give two control schemes to achieve elastic consensus of the system. However, the consensus still has bounded error. At each discrete time, the state value of the agent will be updated. Whether to broadcast it will be decided according to whether the triggered conditions are met: save the last broadcast value as an auxiliary state value; compare it with the updated state value; and trigger if the difference is greater than the threshold value. The author used the modified MSR algorithm and event-triggered mechanism to achieve consensus with bounded error, under the condition that the robustness of relevant topology is satisfied.

Under the event-triggered mechanism, the system cannot achieve accurate consensus and must leave an upper bound of error *c* that grows exponentially with the number of normal agents. One can set the triggering parameter $c_{0}$ by 0 to achieve error free consensus, but the number of triggers will increase. When t→∞, the event-triggered mechanism will lose its effect due to attenuation. The final conclusion is almost the same as that in [82]. Consider reducing the conservatism of the conclusion by adjusting the update structure. Since the update structure reduces unnecessary state updates, under the same error *c*, the number of system triggers will be less, but the convergence speed will be slower. In short, the accuracy of event-triggered mechanism and consensus cannot be reconciled well in the context of MSR algorithm. Compared with periodic sampling control, event triggering reduces the number of information exchanges. However, due to the existence of triggering parameters, there is always an error in the consensus state value. At the same time, for the threshold-triggered mechanism, it is also necessary to consider the balance between the convergence performance and the communication frequency caused by the threshold value. Ref. [85] proposes a sliding-mode control method, which only makes periodic judgment when the state is measurable, does not need continuous triggering condition detection and has better economic benefits, which also belongs to a feasible improvement direction.

Ref. [86] focuses on the observation of the state rather than screening. They achieve the uniform ultimate boundedness of the system ouput. In consideration of malicious agent attacks and bounded sensor errors, it designs an observer so that the outputs of all followers in a heterogeneous system converge successfully to a dynamic convex hull composed of the outputs of complex leaders.

For a heterogeneous system with leaders and attackers:

(36) $$\left\{\begin{array}{c} {\dot{x}}_{i}=A_{i}x_{i}+B_{i}u_{i},\\ y_{i}=C_{i}x_{i} \end{array},\;i\in F,\right.\left\{\begin{array}{c} {\dot{x}}_{l}=Sx_{l},\\ y_{l}=Rx_{l} \end{array},\;l\in \bar{L},\right.\left\{\begin{array}{c} {\dot{x}}_{k}=f_{k}\left(x_{k}\right),\\ y_{k}=C_{k}x_{k} \end{array},\;k\in \bar{A},\right.$$

where $F,\;\bar{L},\;\bar{A}$ are respectively a collection of followers, leaders and attackers. The controller is

(37) $$\begin{array}{ccc} u_{i} & = & K_{i}{\bar{x}}_{i}+H_{i}{\bar{z}}_{i}-H_{i}{\hat{d}}_{i}, \end{array}$$

(38) $$\begin{array}{ccc} {\dot{\bar{z}}}_{i} & = & F_{i}{\bar{z}}_{i}+G_{i}{\bar{e}}_{yi}, \end{array}$$

where ${\bar{e}}_{yi}=\sum_{j\in F}a_{ij}\left({\bar{y}}_{j}-{\bar{y}}_{i}\right)+\sum_{l\in \bar{L}}a_{il}\left(y_{l}-{\bar{y}}_{i}\right)+\sum_{k\in \bar{A}}a_{ik}\left(y_{k}-{\bar{y}}_{i}\right)$. ${\bar{x}}_{i}=x_{i}+\delta_{i}$ is the measurable state value considering the sensor error $\delta_{i}$, ${\bar{z}}_{i}$ is the corresponding state compensation value, and ${\hat{d}}_{i}$ is the compensation signal given by the observer. Matrices $K_{i},\;H_{i},\;F_{i}$ and $G_{i}$ are control gains to be designed. By establishing a judgment mechanism to set $a_{ij}$ or $a_{ik}$ to zero, malicious output signals outside the preset range can be effectively excluded from the update calculation. For malicious values within the range, good fault-tolerant control can be achieved through real-time compensation of ${\hat{d}}_{i}$. Based on the internal model principle and output-feedback control, the following observer scheme can obtain the compensation signal ${\hat{d}}_{i}$:

(39) $$\begin{array}{ccc} {\dot{\hat{x}}}_{i} & = & A_{i}{\hat{x}}_{i}+B_{i}K_{i}{\hat{x}}_{i}+B_{i}H_{i}{\hat{z}}_{i}+B_{i}H_{i}\omega_{i}, \end{array}$$

(40) $$\begin{array}{ccc} {\dot{\hat{z}}}_{i} & = & F_{i}{\hat{z}}_{i}+G_{i}{\hat{e}}_{yi}, \end{array}$$

(41) $$\begin{array}{ccc} {\dot{\hat{\delta }}}_{i} & = & -\left(A_{i}+B_{i}K_{i}\right)\theta_{i}-B_{i}H_{i}\omega_{i}, \end{array}$$

(42) $$\begin{array}{ccc} {\dot{\hat{d}}}_{i} & = & F_{i}{\hat{d}}_{i}-G_{i}C_{i}{\hat{\delta }}_{i}, \end{array}$$

where $\theta_{i}={\bar{x}}_{i}-{\hat{x}}_{i}-{\hat{\delta }}_{i}$, $\omega_{i}={\bar{z}}_{i}-{\hat{z}}_{i}-{\hat{d}}_{i}$, ${\hat{y}}_{i}=C_{i}{\hat{x}}_{i}$, ${\hat{e}}_{yi}=\sum_{j\in F}a_{ij}\left({\hat{y}}_{j}-{\hat{y}}_{i}\right)+\sum_{l\in L}a_{il}\left(y_{l}-{\hat{y}}_{i}\right)$.

The introduction of term $B_{i}H_{i}\omega_{i}$ in Equation (39) makes the real-time feedback adjustment of the observer more flexible, Since $\omega_{i}$ is only related to ${\bar{e}}_{yi}$, this term can change dramatically with the change of sensor error and attacker’s behavior. ${\hat{d}}_{i}$ is mainly affected by $\omega_{i}$ and $\theta_{i}$. The former can be considered as an elastic index to measure the propagation of error ${\bar{e}}_{yi}$ in the communication network, while the latter can reflect the observation accuracy of state variables and sensor errors. The introduction of the above parameters makes it have better compensation effects than the traditional observer [87].

One can get the closed-loop error equation of the system by defining ${\vec{x}}_{i}={\left[{\hat{x}}_{i}^{T},{\hat{z}}_{i}^{T}\right]}^{T}$ and combining the augmented output regulation equation. Then, by proving the stability of the error system, it can be proved that the output feedback control protocol can make the followers’ output values converge to a dynamic convex hull composed of the leader’s output value. In addition, the design of the parameters requires the spectral information of the adjacency matrix, so the protocol is not fully distributed.

At present, there are two main coping strategies for Byzantine attacks: identify malicious agents and move out of the topology [88,89] or design an elastic control structure to maintain the system feature quantity within a tolerable range in the presence of malicious nodes. Refs. [82,84] belong to the latter because the MSR algorithm does not have a detection function. In addition, since the state values are directly screened out, it is equivalent to malicious nodes being invisibly moved out of the topology, which will adversely affect the overall connectivity of the network. For small-scale network topology, the scheme of repairing by real-time compensation is more ideal.

Refs. [90,91] try to improve the algorithm by embedding an input observer. However, this method needs to know the total number of agents attacked and global information such as topology in advance. Ref. [92] breaks through the above limitations and gives a fully distributed observer based on local information for isomorphic systems. The breakthrough of the research still lies in proposing more efficient detection schemes, writing more intelligent screening algorithms and designing more versatile distributed observers.

In addition, refs. [93,94] study the high-order discrete- and continuous-time systems under attack, respectively, by using the robustness of topology. Ref. [95] adopts a robust control scheme based on game theory to achieve elastic consensus. Refs. [96,97] select the weighted MSR algorithm to complete the tracking control of any reference value. Ref. [98] then discusses the security consensus of Byzantine attacks through trusted nodes and also considers setting up decision nodes to avoid malicious values while making specific quantities that tend to be leaders outside the convex hull. Ref. [99] studies the left reversibility of the structure by using the concept of topology node separation, which makes it possible to design communication topology and then realize accurate attack detection. Ref. [90] regards a linear network with abnormal agents as a linear system with sparse actuator anomalies and observes the state with a decoder. Ref. [100] explains the observability of the state of the problem system by using the orthogonal complement matrix, successfully distinguishing the fault agent from the malicious agent, and applying the switched gradient descent algorithm to reduce the computational complexity of the traditional state observation method.

In order to give a clear survey, we summarize the above work in Table 4 according to methodologies, Byzantine attack type, centralized, scalability and references.

### 3.4. Relevant Application Scenarios

In some fields closely related to MASs, the above resilient control algorithms are gradually deployed. For example, in the field of intelligent transportation systems, ref. [101] develops a distributed control strategy based on event-triggered mechanism to deal with deception attacks on the sensor–controller channel and achieves the stability of the vehicle platoon system. Ref. [102] designs a resilient controller composed of observers using sliding mode and adaptive estimation theory, focusing on the detection of the vehicle platoon system under DoS attack. In the field of smart grid, ref. [103] establishes a distributed observer structure based on the principle of consensus control, so as to detect FDI attacks in Distributed Generation Units and isolate infected information channels in DC microgrids. Ref. [104] explores the security control of modern power generation systems under DoS attack and proposes an adaptive resilient control protocol based on event-triggered communication scheme. It applies Lyapunov–Krasovskii functional theory to prove the exponential stability of the smart grid system. More relevant studies can be found in [105]. In the field of sensor networks, ref. [106] discusses the $H_{\infty }$ observation problem under the two-channel FDI attacks. It constructs a distributed observation model against the attack on the basis of the sensor’s own information and neighbors’ information. In the field of multi-robot systems, ref. [107] provides a distributed switching control protocol based on the consensus control theory for DoS attacks and deception attacks. On the one hand, it gives a coordination-free consensus protocol to adjust the weight of each robot under deception attack. On the other hand, based on the control theory of the leader-following system, it converts the robot compromised by DoS attack into a sub-robot following the specific leader.

**Remark** **1.** *It is worth mentioning that all the works reviewed in this survey are gathered in a systematic way: first connecting keywords such as DoS attacks and Byzantine attacks on Google scholar. Then, the reference list of the relevant articles were obtained, followed by narrowing and refining the searching results by year, authors and, finally, source type.*

## 4. Conclusions and Future Directions

In conclusion, we have provided a survey regarding some recent developments on resilient consensus control of MASs. To sum up, for the security consensus of MAS under network attack, there are two main solutions: designing elastic control structure or anomaly observer. It involves a wide range of research fields, such as adaptive control, feedback control, robust control for controller design, stochastic process theory and probability statistics knowledge for attack modeling, and $H_{\infty }$-control theory and optimization methods for system optimization. Some screening algorithms and judgment algorithms in the computer field can even be applied. The main thinking directions for different cyber attacks can be summarized as the following: for DoS attacks with communication interruption, the key is how to intervene in the control of MASs during the period of network paralysis, so that it will not have irreparable consensus deviation. For example, building a tighter topology or a more accurate state observer is a good method. Since deception attacks that tamper information involve data operations, we can choose from schemes such as detecting and moving out of topology or constructing observers and compensators to compensate the error value in real time; For the Byzantine attack that implements induced confusion, it is necessary to design a better malicious agent screening algorithm or repair scheme.

However, the survey is by no means complete. Note that there are still many interesting and yet critical issues concerning MASs under cyber attacks that deserve further study, even though a variety of efficient tools have been successfully developed to solve various challenging problems in this active research field. Some interesting yet important future research issues are provided as follows.

1. Since the effectiveness of network attacks is often accompanied by the appearance of physical faults, an interesting problem is to study network security issues together with fault detection, removal and isolation or integrating other abnormal work issues to improve the practicability of the conclusions, such as saturation problems, measurement noise, communication delay, quantization errors and parameter uncertainty. Especially when complex situations such as mismatched disturbances and multiple time delays are involved, some of the elastic control structures obtained for specific models need to be converted into data-driven ones. Otherwise, it is difficult to play its role. When the detection mechanism confuses faults and attacks, the system may crash. Therefore, the screening methods of the two also belong to the feasible scope of discussion.
2. In the consideration of improving the existing research, another interesting topic is to optimize the control structure, improve the attack stochastic model, achieve more accurate consensus conclusions, relax the theoretical assumptions of the original system and improve the performance of the trigger structure. The further relaxation of the assumptions on the topology network and communication environment is beneficial to the compatibility of the elastic control technology.
3. In the case of actual application, an elastic control scheme need to be designed and modified based on the site situation, so as to boost the feasibility of the excellent theory. For example, in reality, the event-triggered mechanism is often less reliable than the periodic sampling control within the allowable range of communication costs due to noise interference, data clutter, processor performance, sensor sensing abnormalities caused by external factors and actuator failures. Compared with information systems, the control parameter requirements applied to industrial systems are often more stringent because of their high risks.

## Figures and Tables

**Table 1:** Classification of common cyber attacks of MASs.

Limitations of information interaction level	DoS attack, Byzantine attack, spoofing attack, repay attack, police attack, communication attack
Limitations of system level	Actuator attack

**Table 2:** Rencent Work on DoS Attacks.

Control Protocols	DoS Type	Centralized	Scalability	Results	References
Recursive Kalman fusion estimator	Stochastic	Fully	Low	Bounded mean square error	[27]
Event-triggered without observer	Deterministic	Moderate	Low	Leader-following global consensus	[31]
Event-triggered with node-based observer	Stochastic	Moderate	High	Leader-following exponential consensus	[30,40]
Event-triggered with edge-based observer	Stochastic	Moderate	High	State consensus	[38]
Event-triggered with optimization algorithm	Periodic	Moderate	Low	exponential consensus	[48]
Optimized $H_{\infty }$ controller	Hidden and Stochastic	Moderate	Low	σ-error mean square stability	[47]
Distributed low-gain controller	Stochastic	Moderate	Low	Leader-following semi-global consensus	[37]
Predictor-based controller	Deterministic	Moderate	Moderate	Closed-loop stability	[49]

**Table 3:** Recent Work on Spoofing Attacks.

Methodologies	Attack Location	Spoofing Attack Type	Centralized	Scalability	References
Impulse control	Sensor–controller channel	Bernouli distributed FDI	Moderate	High	[64,66,68]
Impulse control	Controller–actuator channel	Bernouli distributed FDI	Moderate	High	[65,66,67]
Secure control	Neighbor information channel	F-local type	Moderate	High	[70,71,73]
Distributed filter with adaptive compensator	Neighbor information channel	Stochastic and bounded	Fully distributed	Low	[69]
Kalman filter based estimator	Information channel	Conditional probability FDI	Moderate	Low	[60]
Neural network based observer	Information channel	White noise based FDI	Moderate	Moderate	[77]
Event-trigger output feedback controller	Neighbor information channel	Bernouli distributed FDI	Moderate	High	[76]

**Table 4:** Recent Work on Byzantine Attacks.

Methodologies	Byzantine Attack Type	Centralized	Scalability	References
MSR algorithm extensions	False data injection	Moderate	High	[85,90,91,92]
Set up trusted decision-making agents	False data injection	Moderate	Moderate	[88,89]
Elastic control	False data injection	Moderate	Moderate	[82,84,86]

## Data Availability

No new data were created or analyzed in this study. Data sharing is not applicable to this article.

## References

[1] Li Y., Xu F., Xie G.Q. (2018). Review of the development and application of multi-agent technology. Comput. Eng. Appl..

[2] Mou Z., Liu B. (2019). Design of a heterogeneous multi-platform sensor management and intelligent control system. J. Command. Control.

[3] Wang J., Deng X., Guo J., Luo Y., Li K. (2022). A fully distributed anti-windup control protocol for intelligent-connected electric vehicles platooning with switching topologies and input saturation. IEEE/ASME Trans. Mechatron..

[4] Guo J., Li L., Wang J., Li K. (2022). Cyber-physical system-based path tracking control of autonomous vehicles under cyber-attacks. IEEE Trans. Ind. Inf..

[5] Fortino G., Russo W., Savaglio C. Agent-oriented Modeling and Simulation of IoT Networks. Proceedings of the Federated Conference on Computer Science and Information Systems (FedCSIS).

[6] Mascardi V., Weyns D. Engineering Multi-agent Systems Anno 2025. Proceedings of the 6th International Workshop on Engineering Multi-Agent Systems (EMAS).

[7] Kampik T., Amaral C.J., Hubner J.F. Developer Operations and Engineering Multi-agent Systems. Proceedings of the 9th International Workshop on Engineering Multi-Agent Systems (EMAS).

[8] Li Y., Tang C., Peeta S., Wang Y. (2019). Nonlinear consensus-based connected vehicle platoon control incorporating car-following interactions and heterogeneous time delays. IEEE Trans. Intell. Transp. Syst..

[9] Shen Y., Wang X., Han S., Chen L., Wang F. (2019). Agent-based technology in intelligent vehicles and drivin: State-of-the-art and prospect. J. Command. Control.

[10] Pasqualetti F., Bicchi A., Bullo F. (2012). Consensus computation in unreliable networks: A system theoretic approach. IEEE Trans. Intell. Transp. Syst..

[11] Zhang T., Li Z. Resilient Network-level Design of Leader-follower Multi-agent Systems Against DoS Attacks. Proceedings of the 39th Chinese Control Conference (CCC).

[12] Ma L.F., Wang Z.D., Yuan Y. Consensus Control for Nonlinear Multi-Agent Systems Subject to Deception Attacks. Proceedings of the 22nd International Conference on Automation & Computing (ICAC).

[13] Su L.L., Vaidya N. Multi-Agent Optimization in the Presence of Byzantine Adversaries: Fundamental Limits. Proceedings of the American Control Conference (ACC).

[14] Franze G., Tedesco F., Famularo D. (2021). Resilience Against Replay Attacks: A Distributed Model Predictive Control Scheme for Networked Multi-Agent Systems. IEEE/CAA J. Autom. Sin..

[15] Sizkouhi A., Rahimifard M., Selmic R. Covert Attack and Detection Through Deep Neural Network on Vision-Based Navigation Systems of Multi-Agent Autonomous Vehicles. Proceedings of the 2022 IEEE International Conference on Systems, Man, and Cybernetics (SMC).

[16] Wang S., Zheng S., Zhao C., Jian H., Li H. Formation control of nonlinear multi-agent systems with actuator and communication attacks. Proceedings of the 40th Chinese Control Conference (CCC).

[17] Ahmadzadeh M., Ahmadi M., Babahaji M., Sharifi I. Resilient Consensus in Double-Integrator Systems with Switching Networks Facing Smart Attacks. Proceedings of the 7th International Conference on Robotics and Mechatronics (ICRoM), Sharif Univ Technol.

[18] Gulzar M.M., Rizvi S.T.H., Javed M.Y., Munir U., Asif H. (2018). Multi-agent cooperative control consensus: A comparative review. Electronics.

[19] Shi P., Yan B. (2021). A survey on intelligent control for multi-agent systems. IEEE Trans. Syst. Man Cybern. Syst..

[20] Duo W., Zhou M.C., Abusorrah A. (2022). A survey of cyber attacks on cyber physical systems: Recent advantages and challenges. IEEE/CAA J. Autom. Sin..

[21] Wu J., Peng C., Yang H., Wang Y. (2022). Recent advances in event-triggered security of networked systems: A survey. Int. J. Syst. Sci..

[22] Ding L., Yan G. (2020). A survey of the security issues and defense mechanisms of multi-agent systems. CAAI Trans. Intell. Syst..

[23] Wang J., Wen G., Duan Z. (2022). Distributed anti-windup consensus control of heterogeneous multi-agent systems over Markovian randomly switching topologies. IEEE Trans. Autom. Control.

[24] Wang J., Guo J., Luo Y., Li K., Zheng H. (2023). Design of switching controller for connected vehicles platooning with intermittent communication via mode-dependent average dwell-time approach. IEEE Internet Things J..

[25] Wang J., Duan Z. (2021). A performance region-based approach to the *H*_∞_ leader-following consensus of nonlinear multiagent systems. Int. J. Robust Nonlinear Control.

[26] Persis C.D., Tesi P. Resilient control under denial-of-service. Proceedings of the 19th IFAC Congress.

[27] Chen B., Ho D.W.C., Zhang W.A., Yu L. (2019). Distributed dimensionality reduction fusion estimation for cyber-physical systems under DoS attacks. IEEE Trans. Syst. Man Cybern. Syst..

[28] Ruan M., Gao H., Wang Y. (2019). Secure and privacy-preserving consensus. IEEE Trans. Autom. Control.

[29] Cardenas A.A., Amin S., Sastry S. Secure control: Towards survivable cyber-physical systems. Proceedings of the 28th International Conference on Distributed Computing Systems Workshops.

[30] Feng Z., Hu G. (2020). Secure cooperative event-triggered control of linear multiagent systems under DoS attacks. IEEE Trans. Control. Technol..

[31] Xu Y., Fang M., Shi P., Wu Z.G. (2020). Event-based secure consensus of mutiagent systems against DoS attacks. IEEE Trans. Cybern..

[32] Lee P., Clark A., Bushnell L., Poovendran R. (2014). A passivity framework for modeling and mitigating wormhole attacks on networked control systems. IEEE Trans. Autom. Control.

[33] Ding D., Wang Z., Han Q., Wei G. (2018). Security control for discrete-time stochastic nonlinear systems subject to deception attacks. IEEE Trans. Syst. Man Cybern.-Syst..

[34] Shames I., Teixeira A.M.H., Sandberg H., Johansson K.H. (2011). Distributed fault detection for interconnected second-order systems. Automatica.

[35] Wang J., Li Y., Duan Z., Zeng J. (2022). A fully distributed robust secure consensus protocol for linear multi-agent systems. IEEE Trans. Circuits Syst. II Express Briefs.

[36] Foroush H.S., Martinez S. On event-triggered control of linear systems under periodic denial-of-service jamming attacks. Proceedings of the IEEE 51st Annual Conference on Decision and Control.

[37] Du S., Yan Q., Gao Y., Wang C. Secure consensus of multiagent systems with input saturation and DoS attacks. Proceedings of the 39th Chinese Control Conference (CCC).

[38] Xu Y., Fang M., Wu Z.G., Pan Y.J., Chadli M., Huang T. (2020). Input-based event-triggering consensus of multiagent systems under denial-of-service attacks. IEEE Trans. Syst. Man Cybern.-Syst..

[39] Zhang K., Wang D., Lv Y. (2020). Review of distributed event-trigger control in multi-agent system. J. Nanjing Univ. Inf. Sci. Technol. (Nat. Sci. Ed.).

[40] Dimarogonas D.V., Frazzoli E., Johansson K.H. (2012). Distributed event-triggered control for multi-agent systems. Trans. Autom. Control.

[41] Tabuada P. (2007). Event-triggered real-time scheduling of stabilizing control tasks. IEEE Trans. Autom. Control.

[42] Gao L., Deng S., Ren W. (2019). Differentially private consensus with an event-triggered mechanism. IEEE Trans. Control. Netw. Syst..

[43] Feng Z., Wen G., Hu G. (2017). Distributed secure coordinated control for multiagent systems under strategic attacks. IEEE Trans. Cybern..

[44] Yang Y., Li Y., Yue D. (2020). Event-trigger-based consensus secure control of linear multi-agent systems under DoS attacks over multiple transmission channels. Sci. China-Inf. Sci..

[45] Yi X., Liu K., Dimarogonas D.V., Johansson K.H. (2019). Dynamic event-triggered and self-triggered control for multi-agent systems. IEEE Trans. Autom. Control.

[46] Cheng T., Kan Z., Klotz J. (2017). Event-Triggered Control of Multiagent Systems for Fixed and Time-Varying Network Topologies. IEEE Trans. Autom. Control.

[47] Wu Z., Xu Y., Lu R., Wu Y., Huang T. (2018). Event-triggered control for consensus of multiagent systems With fixed/switching topologies. IEEE Trans. Syst. Man Cybern.-Syst..

[48] Cheng Z., Yue D., Hu S., Ge H., Chen L. (2020). Distributed event-triggered consensus of multi-agent systems under periodic DoS jamming attacks. Neurocomputing.

[49] Feng S., Tesi P. (2017). Resilient control under denial-of-service: Robust design. Automatica.

[50] Cai B., Zhang L., Shi Y. (2020). Control synthesis of hidden semi-Markov uncertain fuzzy systems via observations of hidden modes. IEEE Trans. Cybern..

[51] Feng Z., Hu G., Wen G. (2016). Distributed consensus tracking for multi-agent systems under two types of attacks. Int. J. Robust Nonlinear Control.

[52] Feng Z., Hu G. Distributed tracking control for multi-agent systems under two types of attacks. Proceedings of the 19th IFAC World Congress.

[53] Ding D., Wang Z., Ho D.W.C., Wei G. (2017). Observer-based event-triggering consensus control for multiagent systems with lossy sensors and cyber-attacks. IEEE Trans. Cybern..

[54] Garcia E., Cao Y., Casbeer D.W. (2014). Decentralized event-triggered consensus with general linear dynamics. Automatica.

[55] Xu W., Ho D.W.C., Zhong J., Chen B. (2019). Event/self-triggered control for leader-following consensus over unreliable network with DoS attacks. IEEE Trans. Neural Netw. Learn. Syst..

[56] Senejohnny D., Tesi P., De Persis C. Self-triggered coordination over a shared network under denial-of-service. Proceedings of the 54th IEEE Conference on Decision and Control.

[57] Shi M. (2019). Research on Network Security and Optimization of Control System Based on Game Theory. Master’s Thesis.

[58] Singh M.P. Cyber security as an application domain for multiagent systems. Proceedings of the 14th International Conference on Autonomous Agents and Multiagent Systems (AAMAS).

[59] Choi I.S., Hong J., Kim T.W. (2020). Multi-agent based cyber attack detection and mitigation for distribution automation system. IEEE Access.

[60] Zuo Z., Cao X., Wang Y. (2020). Security control of multi-agent systems under false data injection attacks. Neurocomputing.

[61] Pang Z., Liu G. (2012). Design and implementation of secure networked predictive control systems under deception attacks. IEEE Trans. Control. Technol..

[62] Liu Y., Ning P., Reiter M.K. (2011). False data injection attacks against state estimation in electric power grids. ACM Trans. Inf. Syst..

[63] Deng R., Liang H. (2019). False data injection attacks with limited susceptance information and new countermeasures in smart grid. IEEE Trans. Ind. Inform..

[64] Wu S., Guo Z., Shi D., Johansson K.H., Shi L. Optimal innovation-based deception attack on remote state estimation. Proceedings of the 2017 American Control Conference.

[65] Li H., Wu Y., Chen M. (2021). Adaptive fault-tolerant tracking control for discrete-time multiagent systems via reinforcement learning algorithm. IEEE Trans. Cybern..

[66] Mustafa A., Modares H. (2020). Attack analysis and resilient control design for discrete-time distributed multi-agent systems. IEEE Robot. Autom. Lett..

[67] He W., Mo Z., Han Q., Qian F. (2020). Secure impulsive synchronization in Lipschitz-type multi-agent systems subject to deception attacks. IEEE-CAA J. Autom. Sin..

[68] He W., Gao X., Zhong W., Qian F. (2018). Secure impulsive synchronization control of multi-agent systems under deception attacks. Inf. Sci..

[69] Huang X., Dong J. (2020). Reliable Leader-to-Follower Formation Control of Multiagent Systems Under Communication Quantization and Attacks. IEEE Trans. Syst. Man Cybern.-Syst..

[70] Fu W., Qin J., Shi Y., Zheng W.X., Kang Y. (2020). Resilient consensus of discrete-time complex cyber-physical networks under deception attacks. IEEE Trans. Ind. Inform..

[71] LeBlanc H.J., Zhang H., Koutsoukos X., Sundaram S. (2013). Resilient asymptotic consensus in robust networks. IEEE J. Sel. Areas Commun..

[72] Li Y., Shi L., Chen T. (2018). Detection against linear deception attacks on multi-sensor remote state estimation. IEEE Trans. Control. Netw. Syst..

[73] Li Y., Fang H., Chen J. (2020). Anomaly detection and identification for multiagent systems subjected to physical faults and cyberattacks. IEEE Trans. Ind. Electron..

[74] Hu L., Wang Z., Han Q., Liu X. (2018). State estimation under false data injection attacks: Security analysis and system protection. Automatica.

[75] Ding D., Wang Z., Ho D.W.C., Wei G. (2017). Distributed recursive filtering for stochastic systems under uniform quantizations and deception attacks through sensor networks. Automatica.

[76] Ding D., Wang Z., Wei G., Alsaadi F.E. (2016). Event-based security control for discrete-time stochastic systems. IET Control Theory Appl..

[77] Sargolzaei A., Yazdani K., Abbaspour A., Crane C.D., Dixon W.E. (2020). Detection and mitigation of false data injection attacks in networked control systems. IEEE Trans. Ind. Inform..

[78] Gusrialdi A., Qu Z., Simaan M.A. (2018). Competitive interaction design of cooperative systems against attacks. IEEE Trans. Autom. Control.

[79] Zhang H., Sundaram S. Robustness of information diffusion algorithms to locally bounded adversaries. Proceedings of the 2012 American Control Conference.

[80] Usevitch J., Panagou D. r-Robustness and (r,s)-Robustness of Circulant Graphs. Proceedings of the 2017 IEEE 56th Annual Conference on Decision and Control.

[81] Dibaji S.M., Ishii H., Tempo R. (2018). Resilient randomized quantized consensus. IEEE Trans. Autom. Control.

[82] Usevitch J., Panagou D. (2020). Resilient leader-follower consensus to arbitrary reference values in time-varying graphs. IEEE Trans. Autom. Control.

[83] Yan H. (2019). Research on Distributed Security Mechanism of Multi-Agent System. Master’s Thesis.

[84] Wang Y., Ishii H. (2020). Resilient consensus through event-based communication. IEEE Trans. Control Netw. Syst..

[85] Behera A.K., Bandyopadhyay B., Yu X. (2018). Periodic event-triggered sliding mode control. Automatica.

[86] Zuo S., Lewis F.L., Davoudi A. (2020). Resilient output containment of heterogeneous cooperative and adversarial multigroup systems. IEEE Trans. Autom. Control.

[87] Hamidreza M., Rohollah M., Lewis F.L., Ali D. (2018). Static output-feedback synchronisation of multi-agent systems: A secure and unified approach. IET Control Theory Appl..

[88] Pasqualetti F., Doerfler F., Bullo F. (2013). Attack detection and identification in cyber-physical systems. IEEE Trans. Autom. Control.

[89] Sundaram S., Hadjicostis C.N. (2011). Distributed function calculation via linear iterative strategies in the presence of malicious agents. IEEE Trans. Autom. Control.

[90] Fawzi H., Tabuada P., Diggavi S. (2014). Secure estimation and control for cyber-physical systems under adversarial attacks. IEEE Trans. Autom. Control.

[91] Dibaji S.M., Ishii H. Resilient consensus of double-integrator multi-agent systems. Proceedings of the 2014 American Control Conference.

[92] Xie C., Yang G. (2017). Decentralized adaptive fault-tolerant control for large-scale systems with external disturbances and actuator faults. Automatica.

[93] Dibaji S.M., Ishii H. (2017). Resilient consensus of second-order agent networks: Asynchronous update rules with delays. Automatica.

[94] LeBlanc H.J., Koutsoukos X. (2018). Resilient first-order consensus and weakly stable, higher order synchronization of continuous-time networked multiagent systems. IEEE Trans. Control Netw. Syst..

[95] Vamvoudakis K.G., Hespanha J.P. (2018). Game-theory-based consensus learning of double-integrator agents in the presence of worst-case adversaries. J. Optim. Theory Appl..

[96] Usevitch J., Panagou D. Resilient leader-follower consensus to arbitrary reference values. Proceedings of the 2018 Annual American Control Conference.

[97] Abbas W., Vorobeychik Y., Koutsoukos X. Resilient consensus protocol in the presence of trusted nodes. Proceedings of the 2014 7th International Symposium on Resilient Control Systems.

[98] Abbas W., Laszka A., Koutsoukos X. (2018). Improving network connectivity and robustness using trusted nodes with application to resilient consensus. IEEE Trans. Control Netw. Syst..

[99] Weerakkody S., Liu X., Son S.H., Sinopoli B. (2017). A graph-theoretic characterization of perfect attackability for secure design of distributed control systems. IEEE Trans. Control Netw. Syst..

[100] Lu A., Yang G. (2020). Secure state estimation for multiagent systems with faulty and malicious agents. IEEE Trans. Autom. Control.

[101] Bansal K., Mukhija P. (2022). Event-triggered control of vehicle platoon under deception attacks. Proc. Inst. Mech. Eng. Part D J. Automob. Eng..

[102] Biron Z., Dey S., Pisu P. (2018). Real-Time Detection and Estimation of Denial of Service Attack in Connected Vehicle Systems. IEEE Trans. Intell. Transp. Syst..

[103] Gallo A., Turan M., Nahata P., Boem F., Parisini T., Ferrari-Trecate G. Distributed Cyber-Attack Detection in the Secondary Control of DC Microgrids. Proceedings of the European Control Conference (ECC).

[104] Lu K., Zeng G., Luo X., Weng J., Zhang Y., Li M. (2020). An Adaptive Resilient Load Frequency Controller for Smart Grids With DoS Attacks. IEEE Trans. Veh. Technol..

[105] Huseinovic A., Mrdovic S., Bicakci K., Uludag S. (2020). A Survey of Denial-of-Service Attacks and Solutions in the Smart Grid. IEEE Access.

[106] Song H., Shi P., Zhang W., Lim C., Yu L. (2020). Distributed *H*_∞_ Estimation in Sensor Networks With Two-Channel Stochastic Attacks. IEEE Trans. Cybern..

[107] Lee S., Min B. (2021). Distributed Control of Multi-Robot Systems in the Presence of Deception and Denial of Service Attacks. arXiv.

